# Vector competence of Australian *Aedes aegypti* and *Aedes albopictus* for an epidemic strain of Zika virus

**DOI:** 10.1371/journal.pntd.0007281

**Published:** 2019-04-04

**Authors:** Leon E. Hugo, Liesel Stassen, Jessica La, Edward Gosden, O’mezie Ekwudu, Clay Winterford, Elvina Viennet, Helen M. Faddy, Gregor J. Devine, Francesca D. Frentiu

**Affiliations:** 1 Mosquito Control Laboratory, QIMR Berghofer Medical Research Institute, Brisbane, Queensland, Australia; 2 Institute of Health and Biomedical Innovation, and School of Biomedical Sciences Queensland University of Technology, Brisbane, Queensland, Australia; 3 QIMR Berghofer Histotechnology Facility, QIMR Berghofer Medical Research Institute, Brisbane, Queensland, Australia; 4 Research and Development, Australian Red Cross Blood Service, Brisbane, Queensland, Australia; University of Wisconsin Madison, UNITED STATES

## Abstract

**Background:**

Recent epidemics of Zika virus (ZIKV) in the Pacific and the Americas have highlighted its potential as an emerging pathogen of global importance. Both *Aedes (Ae*.*) aegypti* and *Ae*. *albopictus* are known to transmit ZIKV but variable vector competence has been observed between mosquito populations from different geographical regions and different virus strains. Since Australia remains at risk of ZIKV introduction, we evaluated the vector competence of local *Ae*. *aegypti* and *Ae*. *albopictus* for a Brazilian epidemic ZIKV strain. In addition, we evaluated the impact of daily temperature fluctuations around a mean of 28°C on ZIKV transmission and extrinsic incubation period.

**Methodology/Principal findings:**

Mosquitoes were orally challenged with a Brazilian ZIKV strain (8.8 log CCID_50_/ml) and maintained at either 28°C constant or fluctuating temperature conditions. At 3, 7 and 14 days post-infection (dpi), ZIKV RNA copies were quantified in mosquito bodies, as well as wings and legs, using qRT-PCR, while virus antigen in saliva (a proxy for transmission) was detected using a cell culture ELISA. Despite high body and disseminated infection rates in both vectors, the transmission rates of ZIKV in saliva of *Ae*. *aegypti* (50–60%) were significantly higher than in *Ae*. *albopictus* (10%) at 14 dpi. Both species supported a high viral load in bodies, with no significant differences between constant and fluctuating temperature conditions. However, a significant difference in viral load in wings and legs between species was observed, with higher titres in *Ae*. *aegypti* maintained at constant temperature conditions. For ZIKV transmission to occur in *Ae*. *aegypti*, a disseminated virus load threshold of 7.59 log_10_ copies had to be reached.

**Conclusions/Significance:**

Australian *Ae*. *aegypti* are better able to transmit a Brazilian ZIKV strain than *Ae*. *albopictus*. The results are in agreement with the global consensus that *Ae*. *aegypti* is the major vector of ZIKV.

## Author summary

Zika virus (ZIKV) is a mosquito-borne pathogen that generally causes a mild febrile illness but mostly remains asymptomatic in 50–80% of infections. Infection during pregnancy can cause congenital malformations, notably microcephaly. In adults, it can cause Guillain-Barré syndrome. The recent ZIKV epidemic in the Americas has been linked to the urban vector *Aedes aegypti*. The presence of the species in Australia makes the region vulnerable to emerging mosquito-borne viruses. A mosquito’s competence to transmit a pathogen will depend on both the virus and vector strains. Here, we determine the vector competence of Australian *Ae*. *aegypti* and *Ae*. *albopictus* mosquitoes for a ZIKV epidemic strain, originating from the epicentre of the Brazilian outbreak, under constant and fluctuating temperatures that simulate field environments in Australia. Our results demonstrate that, although both species were susceptible to ZIKV infection, *Ae*. *aegypti* is more likely to transmit virus. Our results may aid in the formulation of public health strategies to mitigate the threat of ZIKV.

## Introduction

Over the past decade, Zika virus (ZIKV) has caused unprecedented epidemics in the Western Pacific and the Americas. ZIKV is a mosquito-borne, single-stranded RNA virus that belongs to the *Flavivirus* genus within the *Flaviviridae* family [1]. First discovered in Uganda in 1947 [2], ZIKV spread from equatorial Africa into Asia in 1960, producing two main genotypes, the African and Asian lineages [3, 4]. Major epidemics of ZIKV have occurred on Yap Island, Federated State of Micronesia [5, 6], French Polynesia [7], some islands in the south and south-west Pacific region [8–11], and most recently Latin America [12–15]. Although 80% of ZIKV infections remain asymptomatic or cause a mild febrile illness [5, 16], recent epidemics have seen more severe disease manifestations, such as microcephaly and central nervous malformations in neonates [17, 18], and Guillain-Barré syndrome in adults [19, 20].

Although ZIKV can be transmitted sexually [21], through blood transfusion [22], and from mother-to-child [23], humans are primarily infected through the bite of infected *Aedes* (*Ae*.) mosquito species [24–28]. In Africa, where it was first isolated from *Ae*. *africanus* [29], ZIKV is mainly transmitted by sylvatic *Aedes* mosquitoes (*Ae*. *furcifer*, *Ae*. *luteocephalus*, *Ae*. *taylori*, *Ae*. *opok*, *Ae dalzieli*) [24, 30]. Initial evidence for human infections implicated *Ae*. *aegypti* in the urban transmission of ZIKV in Africa [26, 31, 32]. In Asia [4, 33, 34] and the Americas [35–37], *Ae*. *aegypti* is considered the main vector for human ZIKV transmission. Although *Ae*. *hensilli* was suspected to be responsible for ZIKV transmission during the Yap outbreak [27], *Ae*. *aegypti* and *Ae*. *polynesiensis* were the main vectors in the French Polynesian outbreak [28]. Recent evidence of vertical transmission of ZIKV in field-collected eggs of *Ae*. *aegypti* from Brazil suggests that, in endemic areas, virus may also be maintained in drought resistant eggs [38]. *Ae*. *albopictus* is an invasive vector which has colonized most of the tropics and subtropics, as well as more temperate regions of the United States and Europe [39]. The high vectorial capacity of *Ae*. *albopictus* for various arboviruses [40–42] places any area colonized by this species at risk of local ZIKV transmission [43, 44]. Considerable variation in ZIKV vector competence, similar to that reported for DENV [45–47], has been observed in both *Ae*. *aegypti* and *Ae*. *albopictus* from across the globe [25, 48–54]. The transmission efficiency of ZIKV is governed by interactions between mosquito strain [25, 53] and virus genotype/strain [45, 53, 55–57]. This variability underscores the importance of evaluating the vector competence of local mosquito populations for ZIKV.

Australia remains at risk of ZIKV introduction due to its close proximity to the Western Pacific, the presence of competent strains of *Ae*. *aegypti* in Queensland [58, 59] and *Ae*. *albopictus* in the Torres Strait [48, 60], and favourable climatic conditions for transmission [61]. Despite 51 reports of imported cases of ZIKV since 2014 (Queensland Government, Australia, accessed 8 October 2018), Australia has not yet reported autochthonous transmission. Previous studies have reported the vector competence of Australian *Ae*. *aegypti* for African, Cambodian and Western Pacific strains [48, 58, 59] and *Ae*. *albopictus* (Torres Strait islands) for Cambodian ZIKV [48, 58, 59]. These studies demonstrated that Australian mosquito strains can be infected and transmit ZIKV; however, large heterogeneity has been observed in the susceptibility of mosquitoes to infection, which may be associated with the origin of the virus strains. There have been no investigations of the vector competence of Australian strains to isolates of ZIKV from South America, despite the continent recording the largest epidemics with a high prevalence of the most severe ZIKV disease manifestations [62]. To assess the public health risk imposed by ZIKV to Australia, we determined the vector competence of local populations of *Ae*. *aegypti* and *Ae*. *albopictus* (Torres Strait Islands) for a strain of ZIKV isolated from a febrile patient from Paraiba state, at the centre of the 2015/2016 Brazil epidemic. In addition to maintaining infected mosquitoes under a standard constant temperature regime, we also used a fluctuating diurnal temperature range (DTR). Our study indicates that *Ae*. *aegypti* has higher relative vector competence than *Ae*. *albopictus*, which may be mediated by a salivary gland barrier to virus transmission in *Ae*. *albopictus* for this ZIKV strain.

## Methods

### Mosquitoes

*Ae*. *aegypti* eggs were obtained from a colony established from *Wolbachia*-free eggs collected from Innisfail, north Queensland, in April 2016 and subsequently maintained in the QIMR Berghofer insectary at 27°C, 70% relative humidity [RH] and 12:12 h lighting with 30 min crepuscular periods. *Ae*. *albopictus* eggs were obtained from a colony established from eggs collected on Hammond Island, Torres Strait, Australia, in July 2014 and subsequently maintained in the QIMR Berghofer insectary. Eggs of both colonies were hatched and larvae were reared at a density of 400 individuals in 3 L of rainwater. Larvae were provided ground TetraMin Tropical Flakes fish food (Tetra, Melle, Germany) *ad libitum*. Pupae were transferred to a container of rainwater inside a 30 × 30 × 30 cm cage (BugDorm, MegaView Science Education Services Co., Taichung, Taiwan) for adult emergence. Adult mosquitoes were provided with 10% sucrose solution on cotton wool pledgets.

### Virus strain

The Brazilian ZIKV strain KU365780 [63] used in this study was isolated from a febrile patient in Joao Pessoa City, Paraiba State, Brazil, 18-05-2015 (provided by the Evandro Chagas Institute, Brazil). Viruses were propagated in C6/36 *Ae*. *albopictus* cells, maintained at 28°C in RPMI-1640 medium (Sigma Life Sciences, USA) supplemented with 10% fetal bovine serum. Following three passages in C6/36 cells, virus stocks were concentrated using Ultracel-100k filters (Amicon, Tullagreen, Cork Ireland) and frozen once at -80°C until further use. Virus stocks were titrated using a Cell Culture Enzyme-linked Immunosorbant Assay (CCELISA) based on the method of Broom et al. [64]. Ten-fold serial dilutions of virus stocks were inoculated on C6/36 (*Ae*. *albopictus*) cells grown in RPMI 1640 supplemented with L-glutamine, 5% heat denatured FBS, 1% penicillin/streptomycin (Gibco Life Technologies, USA) and maintained at 28°C, 5% CO_2_ for 5 days. Monolayers were incubated at 28°C, 5% CO_2_ for 5 days, and cells fixed at -20°C for 1 h in 80% acetone/20% phosphate-buffered saline (PBS). Plates were air-dried and antigen was detected using a 4G4 anti-Flavivirus NS1 monoclonal antibody hybridoma supernatant (1:40 in PBS-Tween), Horseradish peroxidase (HRP-) conjugated goat anti-mouse polyclonal antibody (DAKO, Carpinteria, CA, USA) (1:2000 in PBS-Tween), and 3,3′,5,5′-Tetramethylbenzidine (TMB) Liquid Substrate System for Membranes (Sigma-Aldrich, St. Louis, MO, USA). Staining was observed using an inverted microscope, and cell monolayers that stained blue were scored positive for infection. The 50% cell culture infectious dose (CCID_50_) was determined from titration end points as previously described [65] and expressed as the CCID_50_/ml in C6/36 cells.

### Mosquito *per os* exposure to ZIKV

**Fig 1:**
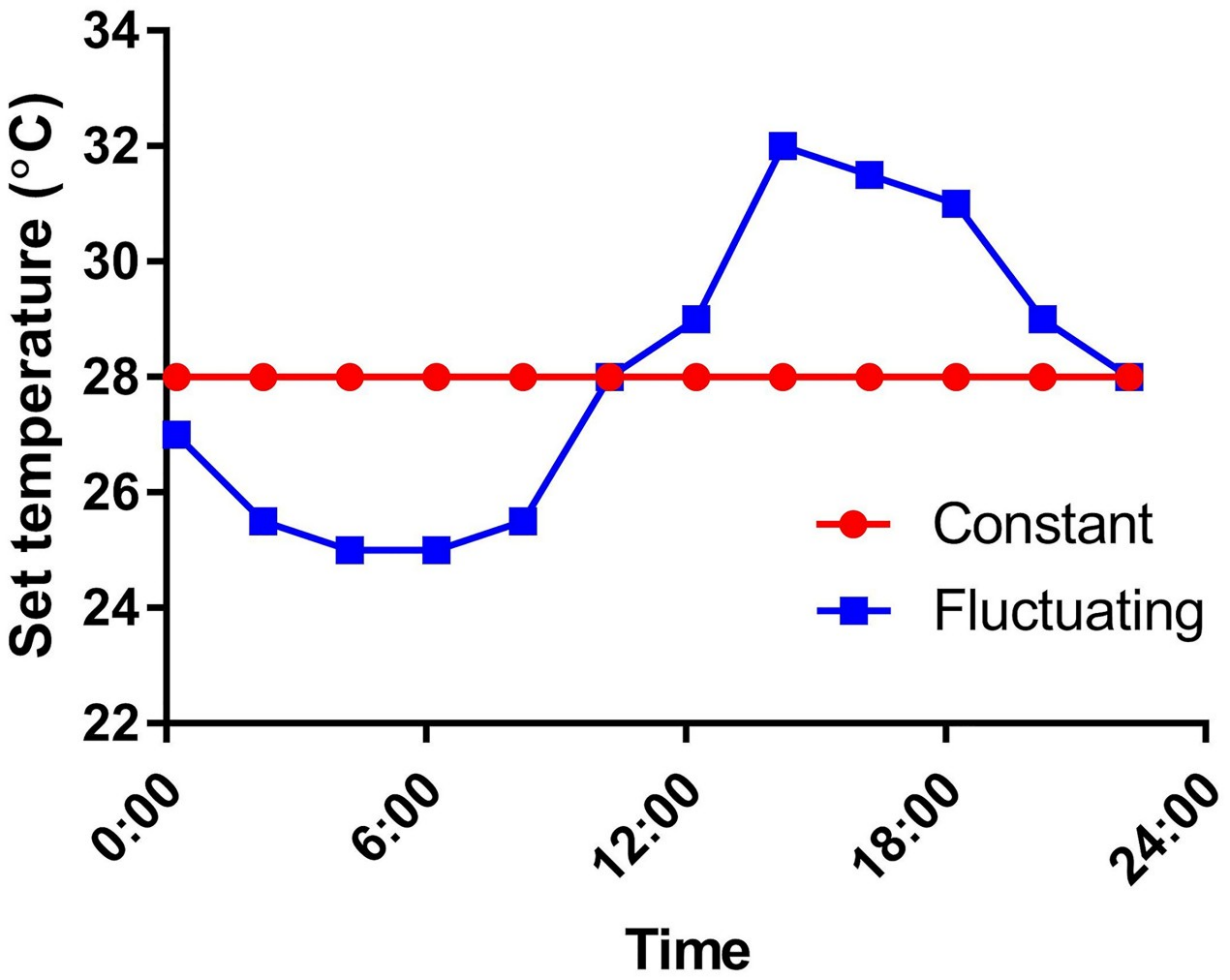
Constant and fluctuating temperature regimes. Temperature set points for the maintenance of mosquitoes in the constant and fluctuating temperature regimes. Relative humidity was set at 75% and a 12:12 h day:night light cycle was used.

Mosquito infection with ZIKV occurred in a Biosafety Level 3 insectary at QIMR Berghofer. An artificial membrane feeding apparatus, fitted with a porcine intestinal membrane, was used to orally challenge adult females (3–5 day old) with a mixture of defibrinated sheep blood (Life Technologies, Mulgrave, VIC, Australia) and virus at a final concentration of 8.8 log CCID_50_/ml (C6/36 cells) for 1 h. Following ZIKV inoculation, mosquitoes were maintained in environmental growth chambers (Panasonic), with either a constant temperature program set to 28°C or a fluctuating (cyclical) temperature program (24.5–32°C) around a mean of 28°C [66] (Fig 1). The temperature treatments are referred to here as “constant” and “fluctuating”, respectively. For both treatments, RH was set to 75% and a 12:12 h day:night lighting program was used.

### Mosquito processing

Twenty mosquitoes were harvested at each of three time points (3, 7 and 14 days) post blood feeding by anaesthetizing the insects with CO_2_ on ice before removing legs and wings. Mosquitoes were gently transferred by their antennae to a glass plate and immobilized on double-sided sticky tape. Saliva was collected by placing the proboscis of each mosquito into a 200 μl pipette tip containing 10 μl of 10% FBS and 10% sugar solution. The insertion of the proboscis into the salivation solution was performed under a dissecting scope and peristaltic movement of the abdomen observed to indicate salivation. Mosquitoes were allowed to salivate for 20 min, after which the contents of the pipette tip were then expelled into a microtube and preserved at -80°C.

### Nucleic acid extraction

Nucleic acids were extracted from individual mosquito bodies or body parts using the High Pure Viral Nucleic Acid Kit (Roche Diagnostics, Mannheim, Germany) according to the manufacturer’s protocol. Briefly, 200 μl Binding Buffer/poly A solution was added to each 2 ml screw cap vial containing the individual mosquito body or body parts. The samples were homogenized by shaking the tubes, containing zirconium silica glass beads (Daintree Scientific, St Helens, TAS, Australia), using a MiniBeadbeater-96 (Biospec Products, Bartlesville, Oklahoma, USA) for 90 s. Following the addition of 50 μl of Proteinase K, nucleic acids were extracted as per the manufacturer’s instructions, and eluted in 50 μl of RNAse/DNase-free Ultrapure water (Invitrogen). Samples were frozen at -80°C until further analysis.

### Quantitative RT-PCR to detect ZIKV

To determine the absolute number of ZIKV copies in each mosquito body or body part, a control plasmid, containing a cloned copy of the targeted ZIKV gene fragment (nucleotides 835 to 911, Genbank accession number EU545988), was constructed. Viral RNA was extracted using the QIAamp Viral RNA Mini Kit (Qiagen, Germany), and cDNA synthesized using the SuperScript III Reverse Transcriptase kit (Invitrogen, Thermo Fisher Scientific, USA) according to the manufacturer’s protocol. The targeted ZIKV fragment was amplified using CloneAmp HiFi PCR Premix (Takara, Clontech Laboratories, USA), and cloned into the pUC19 plasmid vector (Genscript, New Jersey, United States) using the In-Fusion Cloning Kit (Takara, Clontech Laboratories, USA) as described by the manufacturer. The presence of the insert DNA was confirmed by nucleotide sequencing. For quantitative RT-PCR analysis, the plasmid was linearized by *Eco*RI (Promega, USA) and purified using the Nucleospin Gel and PCR clean-up kit (Macherey-Nagel, Germany). The concentration and purity of the linearized plasmid DNA was determined using the NanoDrop Lite spectrophotometer (Thermo Fisher Scientific, USA). The plasmid copy number was calculated based on the measured DNA concentration and its molecular weight. Plasmid DNA concentrations were confirmed prior to the preparation of a 10-fold serial dilution from 3×10^7^ to 3×10^2^ copies/μl and run in parallel with the samples in all qRT-PCRs.

ZIKV RNA from mosquitoes was amplified by one-step qRT-PCR using the TaqMan Fast Virus 1-Step Master Mix (Applied Biosystems, USA) according to the manufacturer’s protocol, in a Rotor-Gene 6000 Version 1.7.87 system (Corbett Research, NSW, Australia). Primers and probe used in this study have previously been described [6] and were synthesized at Macrogen, Korea. The probe was labelled with FAM and BHQ1 at the 5′ and 3′ ends, respectively. The 20 μl reaction mixture consisted of 1 μl extracted sample, 4 × TaqMan Fast Virus 1-Step Master Mix, 400 nM of each primer, 250 nM of probe and Ultrapure water (Invitrogen, Thermo Fisher Scientific, USA). Reactions were run in triplicate, and a 10-fold serial dilution of linearized control plasmid DNA (3×10^7^ to 3×10^2^ copies/μl), as well as negative controls (without template), were included in each run. The following thermal profile was used: a single cycle of reverse transcription for 5 min at 50°C, reverse transcriptase inactivation and DNA polymerase activation at 95°C for 20 s followed by 40 cycles of 95°C for 3 s and 60°C for 30 s (annealing-extension step). Data were analysed and quantified using the Rotor-Gene 6000 Version 1.7.87 software (Corbett Research, NSW, Australia). To calculate the total number of ZIKV RNA copies present in each mosquito body or body part, the measured ZIKV RNA copy numbers in 1μl were multiplied by the elution volume (i.e., 50 μl). Samples were scored positive for virus if ZIKV amplification occurred in at least two technical replicates and the number of copies was above the limit of detection of the standard curve.

Samples in which ZIKV failed to amplify were classified as negative. The presence of mosquito nucleic acid in negative samples was verified by amplification of the housekeeping genes RpS17 (*Ae*. *aegypti*; Genbank accession number AY927787.2) or RpS7 (*Ae*. *albopictus*; Genbank accession number XM_019671546). qRT-PCR for house-keeping genes were performed using the SuperScript III SYBR Green One-Step qRT-PCR kit (Invitrogen, Life Technologies, USA) as per manufacturer’s recommendations. The reactions were performed in a 10 μL total volume containing SuperScript III RT/Platinum Taq Mix, 2 × SYBR Green Reaction Mix, 200 nM of each RpS17/RpS7 primer (RpS17 F: 5′-TCCGTGGTATCTCCATCAAGCT-3′, R: 5′-CACTTCCGGCACGTAGTTGTC-3′; RpS7 F: 5’-CTCTGACCGCTGTGTACGAT-3’, R: 5’-CAATGGTGGTCTGCTGGTTC-3’), 1 μL of extracted sample and Ultrapure water (Invitrogen, Life Technologies, USA). Cycling was performed using the Rotor-Gene 6000 system (Corbett Research, NSW, Australia) with 1 cycle at 50°C for 5 min and 95°C for 2 min, followed by 40 amplification cycles of 95°C for 15 s, 60°C for 30 s and 72°C for 20 s. Melt curve analysis was performed to analyse the specificity of the reaction.

### CCELISA of blood meals and mosquito saliva

The presence of infectious virus in blood meals and in mosquito saliva samples was determined using CCELISA as described above, with the following modifications. Blood meals were titrated by 10-fold serial dilution on C6/36 (*Ae*. *albopictus*) cells grown in RPMI 1640 supplemented with L-glutamine, 5% heat denatured FBS, 1% penicillin/streptomycin (Gibco Life Technologies, USA) and maintained at 28°C, 5% CO_2_ for 5 days. Mosquito saliva samples were diluted 1:25 in the media described above, supplemented with 0.1% Gibco Amphotericin B (ThermoFisher Scientific, Waltham, MA USA), and used to inoculate duplicate monolayers of C6/36 cells (~90% confluent). Samples were then fixed and stained as described above.

### Immunofluorescence analysis

The legs and wings were removed from mosquitoes and the remaining body was fixed in 4% paraformaldehyde and 0.5% Triton X overnight before mosquitoes were transferred to 70% ethanol. Mosquitoes were dehydrated and embedded in paraffin using standard procedures. Paraffin sections (3–4 μM) were fixed to Menzel Superfrost Plus glass histology slides (Menzel-Gläser, Braunschweig, Germany) and air-dried overnight at 37°C. The sections were dewaxed and rehydrated in a descending alcohol series to water, and antigen retrieval was performed in Dako Target Retrieval solution (pH 9.0) at 100°C for 20 min using a Biocare Medical decloaking chamber. On completion of the cooling cycle, slides were cooled for a further 20 min before being washed in three changes of Tris-buffered saline containing 0.025% Tween 20 (TBS-Tween). The sections were incubated in Background Sniper solution (Biocare Medical, Walnut Creek, CA, USA) plus 1% BSA for 15 min to inhibit nonspecific antibody binding. Excess Background Sniper was removed and slides transferred to an opaque humidified chamber for subsequent incubation steps. Sections were incubated in 4G4 anti-Flavivirus NS1 monoclonal antibody hybridoma supernatant (undiluted) overnight at room temperature in a humidified chamber, washed three times in TBS-Tween, and incubated for 1 h in AlexaFluor-488 conjugated donkey anti-mouse antibody, diluted 1:300 in TBS-Tween. After washing three times in TBS-Tween, sections were counterstained with the fluorescent DNA stain 4',6-diamidino-2-phenylindole (DAPI) for 5 min, washed as above and mounted with coverslips using Dako fluorescent mounting medium. Slides were scanned using an Aperio ScanScope Fl slide scanner (Aperio Techologies, Vista, CA, USA) at a magnification of 20×. Quantitative image analysis was performed as previously described [67].

### Data analysis

Percentage infection (number of positive bodies/total tested), dissemination (number of positive leg/wing samples per total tested), and transmission (number of positive saliva samples/total tested) were calculated at each dpi for each species under fluctuating and constant temperature regimes. Significant differences between percentages were detected using Chi-Square tests. The median and interquartile range (IQR) values were calculated using GraphPad Prism Version 7.00 (GraphPad Software, La Jolla, California USA, 2008). Log-transformed virus titres in mosquitoes with infected bodies and wings and legs were analysed using two-way Analysis of Variance (ANOVAs) as a function of temperature, species, and their interactions, in IBM SPSS Statistics software version 23.0. Differences were considered statistically significant at *p* < 0.05. Receiver Operator Characteristic (ROC) curve analysis was performed for both species to predict threshold disseminated titre at which saliva infection was likely to occur. ROC curve analyses were performed using the pROC package in R version 1.12.1 (May 2018) [68], with samples pooled across days and temperatures for each mosquito species to ensure maximum predictive power. The ZIKV staining density (ratio of ZIKV/DNA positive pixel area) within defined tissues in histological sections was analysed by two-way ANOVA as a function of temperature, days post infection and their interaction using GraphPad Prism Version 8.02. Post hoc comparisons of the main effects of days post infection were performed using Sidak’s multiple comparison test.

## Results

### ZIKV infection, dissemination and transmission

High body infection percentages (number of positive bodies/total mosquitoes tested) were observed for both mosquito species under constant and fluctuating temperature conditions, at all the time points tested (Table 1). The body infection percentage in *Ae*. *aegypti* were 80% (constant) and 75% (fluctuating) at 3 dpi, 65% (constant) and 70% (fluctuating) at 7 dpi, and 70% (constant and fluctuating) at 14 dpi (Table 1). Compared to *Ae*. *aegypti*, higher body infection percentages were observed in the *Ae*. *albopictus* temperature groups at all time points (Table 1). Infection percentages in *Ae*. *albopictus* bodies reached 95% (constant) and 85% (fluctuating) at 3 dpi, 90% (constant and fluctuating) at 7 dpi, and 80% (constant) and 100% (fluctuating) at 14 dpi (Table 1).

**Table 1:** Infection, dissemination and transmission percentages of ZIKV_BR_ in *Ae*. *aegypti* and *Ae*. *albopictus* maintained at a 28°C constant or fluctuating temperature conditions.

		% Infection^1^		% Dissemination^2^		% Transmission^3^	
dpi	Species	Constant	Fluctuating	Constant	Fluctuating	Constant	Fluctuating
3	*Ae*. *aegypti*	80 (17/20)	75 (15/20)	10 (2/20)	10 (2/20)	5 (1/20)	0 (0/20)
	*Ae*. *albopictus*	95 (19/20)	85 (17/20)	15 (3/20)	0 (0/20)	0 (0/20)	0 (0/20)
7	*Ae*. *aegypti*	65 (13/20)	70 (14/20)	60 (12/20)	70 (14/20)	0 (0/20)	0 (0/20)
	*Ae*. *albopictus*	90 (18/20)	90 (18/20)	70 (14/20)	60 (12/20)	10 (2/20)	0 (0/20)
14	*Ae*. *aegypti*	70 (14/20)	70 (14/20)	70 (14/20)	70 (14/20)^4^	60 (12/20)^6^	50 (10/20)^7^
	*Ae*. *albopictus*	80 (16/20)	100 (20/20)	45 (9/20)^5^	100(20/20)^4^^,^^5^	10 (2/20)^6^	10 (2/20)^7^

^1^Percentage mosquitoes containing virus in their bodies (number of positive bodies/total tested).

^2^Percentage mosquitoes containing virus in their wings and legs (number of positive leg/wing samples per total tested).

^3^Percentage mosquitoes containing virus in their saliva (number of positive saliva samples/total tested).

^4, 5, 6 & 7^ Superscript numbers indicate specific comparisons between treatment groups that were significantly different (p-values < 0.05 by Chi-Square).

Disseminated infection percentages in *Ae*. *aegypti* increased from 10% (constant and fluctuating) at 3 dpi, to 60% (constant) and 70% (fluctuating) at 7 dpi, and remained at 70% (constant and fluctuating) thereafter (Table 1). Disseminated infection percentages in *Ae*. *albopictus* were 15% (constant) and 0% (fluctuating) at 3 dpi, 70% (constant) and 60% (fluctuating) at 7dpi, and 45% (constant) and 100% (fluctuating) at 14 dpi, with significant differences at this time interval (Table 1). We also found a significant difference in dissemination percentages between the vector species for the fluctuating temperature regime (Table 1).

At early time points, ZIKV was either generally not detectable in saliva, or transmission percentages were too low to be detected with our sample size. None of the *Ae*. *aegypti* in the fluctuating temperature group were infectious at 3 dpi; however, in the constant temperature group, ZIKV was detected in the saliva of a single *Ae*. *aegypti* mosquito (5% transmission) (Table 1). ZIKV was not detected in the saliva of *Ae*. *albopictus* at 3 dpi (Table 1). At day 7 dpi, no *Ae*. *aegypti* saliva samples were found to contain infectious ZIKV. At the same time point, ZIKV was first detected in the saliva of *Ae*. *albopictus* mosquitoes maintained at constant temperature (10% transmission), but not in the fluctuating temperature group. The ZIKV transmission percentages of *Ae*. *aegypti* were significantly higher than in *Ae*. *albopictus* at 14 dpi, for both temperature conditions (Table 1). Whereas transmission percentages of 60% (constant) and 50% (fluctuating) were observed for *Ae*. *aegypti* at 14 dpi, only 10% (constant and fluctuating) of *Ae*. *albopictus* had infectious saliva at this time point (Table 1).

### Kinetics of ZIKV RNA replication in bodies and wings and legs of *Ae*. *aegypti* and *Ae*. *albopictus* mosquitoes

**Fig 2:**
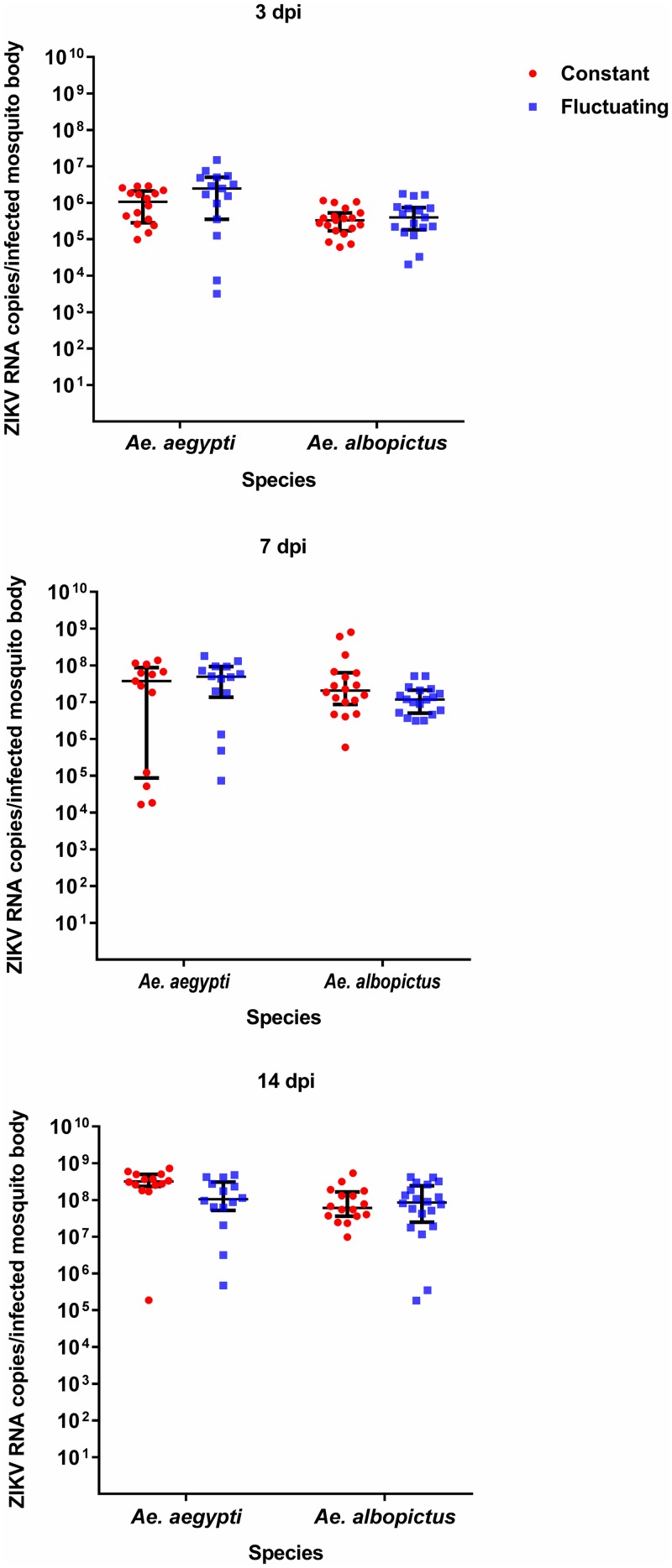
ZIKV RNA copies in *Ae*. *aegypti* and *Ae*. *albopictus* bodies. Comparison of ZIKV RNA copies in the bodies of *Ae*. *aegypti* and *Ae*. *albopictus* maintained at 28°C constant or fluctuating temperature conditions. The amount of ZIKV RNA copies in mosquito bodies were quantified by qRT-PCR at 3, 7 and 14 dpi. Each point on the plot represents an individual mosquito. All plots show the median value ± interquartile range (IQR).

Both species exhibited high viral loads (>10^7^ copies/body) in bodies from 7 dpi in constant and fluctuating temperature groups, which remained at high levels until 14 dpi (Fig 2, S1 Table). No significant differences were observed in viral copy number in bodies between constant and fluctuating temperature regimes (*p* > 0.05). Overall, we found no significant effect of temperature (*p* = 0.718), species (*p* = 0.107), or an interaction between these two factors (*p* = 0.411) on viral load in mosquito bodies. We did find a significant effect of day post-infection (*p* < 0.0005) on virus loads, consistent with the observed increase in body titre across the time points in both species (Fig 2, S1 Table).

**Fig 3:**
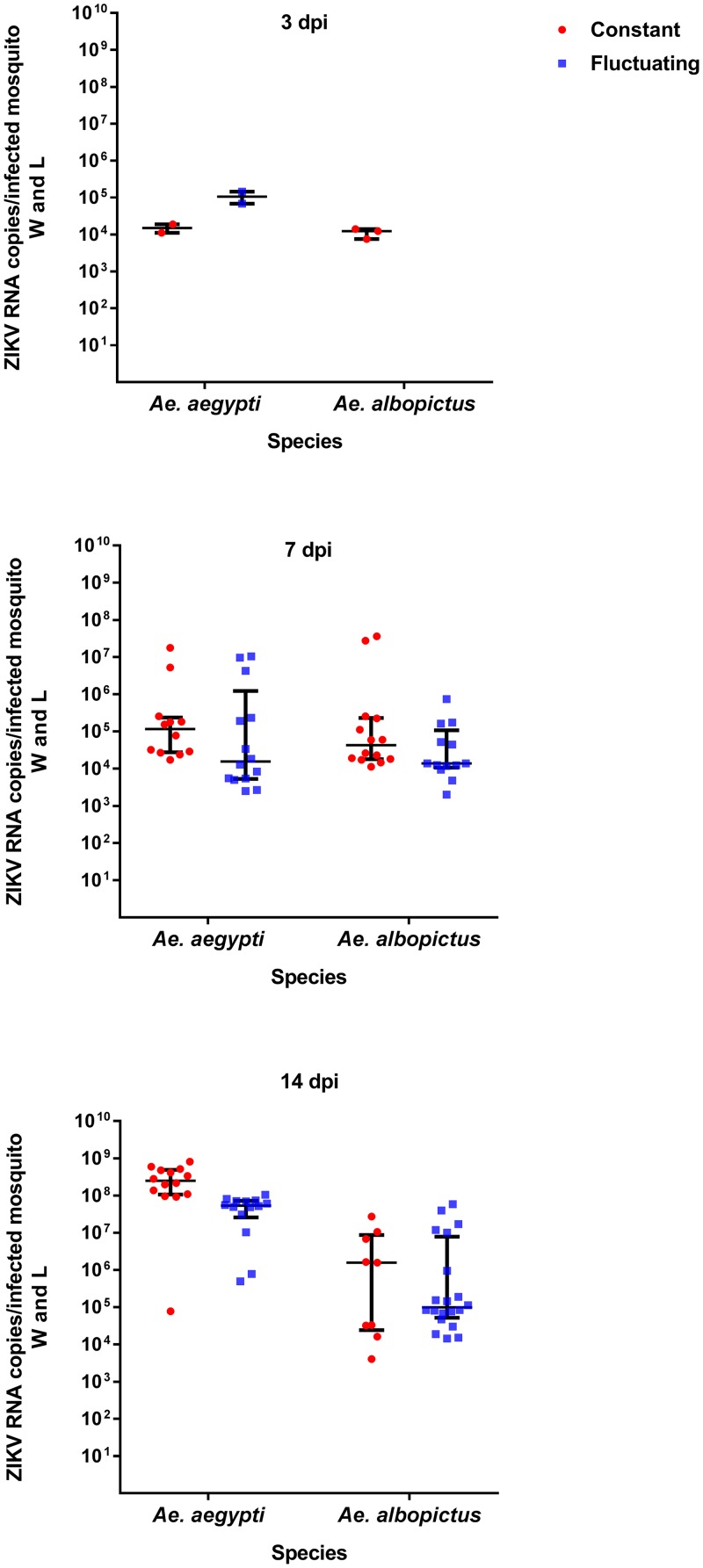
ZIKV RNA copies in *Ae*. *aegypti* and *Ae*. *albopictus* wings and legs. Comparison of ZIKV RNA copies in the wings and legs of *Ae*. *aegypti* and *Ae*. *albopictus* maintained at 28°C constant or fluctuating temperature conditions. The amount of ZIKV RNA copies in mosquito wings and legs were quantified by qRT-PCR at 3, 7 and 14 dpi. Each point on the plot represents an individual mosquito. All plots show the median value ± interquartile range (IQR).

ZIKV RNA was detected in the wings and legs of *Ae*. *aegypti* constant and fluctuating temperature groups at 3 dpi, albeit in only a very few mosquitoes (Fig 3, S2 Table). The median number of RNA genome copies in the wings and legs of both *Ae*. *aegypti* temperature groups increased from 3 dpi and reached its highest level at 14 dpi (>10^7^ copies/mosquito wings and legs) (Fig 3, S2 Table). At early time points (3 dpi), levels of ZIKV RNA were ~10^4^ copies/ wings and legs in *Ae*. *albopictus* mosquitoes maintained at constant temperature. Thereafter, ZIKV RNA levels in *Ae*. *albopictus* marginally increased in both the constant and fluctuating temperature groups until 14 dpi (Fig 3, 7 and 14 dpi, S2 Table). In contrast to *Ae*. *aegypti*, a significantly lower disseminated viral load (*p* < 0.05) was observed in the wings and legs of *Ae*. *albopictus* mosquitoes at day 14 (Fig 3, 14 dpi, S2 Table). Overall, a significant difference in the disseminated viral load was observed between species (Fig 3, S2 Table). Significant effects in disseminated titre due to day (*p* < 0.001), species (*p* = 0.001) and temperature (*p* = 0.032) were identified. The results suggested that exposure to constant versus fluctuating temperature does influence viral disseminated titre, although these effects were only observed at days 3 and 14 post-infection (Fig 3, S2 Table). Furthermore, there was a statistically significant interaction between species and dpi (*p* < 0.001), indicating that disseminated titres differed between species within each of the days post-infection sampled here.

### Localization of ZIKV in mosquito tissue

**Fig 4:**
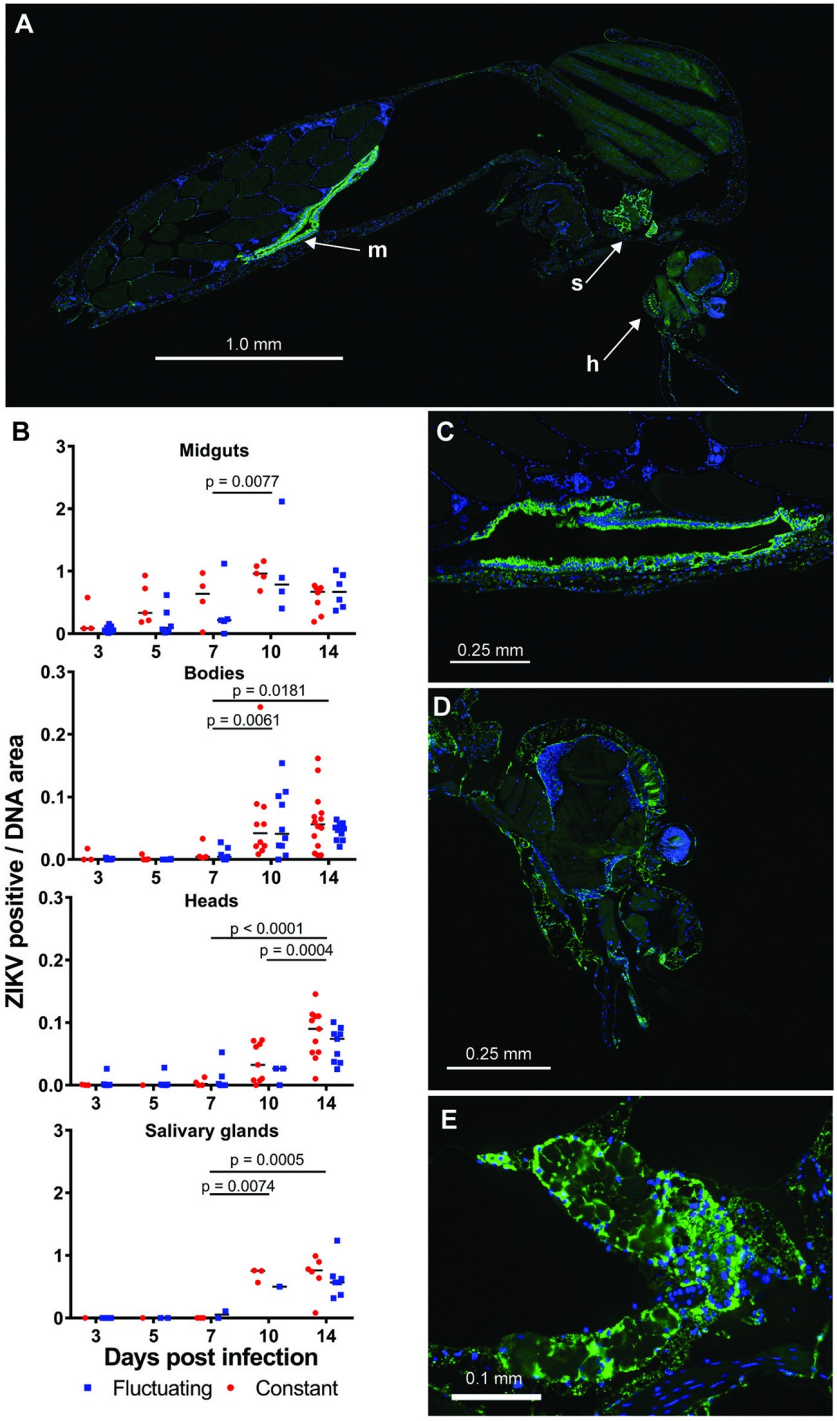
*In vivo* distribution of ZIKV infection in *Ae*. *aegypti* mosquitoes using immunofluorescence assay (IFA) with whole mosquito microscopy. Mosquitoes were examined by IFA for ZIKV by staining with an anti-*Flavivirus* NS1 protein monoclonal antibody (green) and DAPI staining for DNA (blue). (A) An example of a whole mosquito body section showing ZIKV infection in the midgut (m), head (h) and salivary glands (s). (B) Quantification of anti-ZIKV staining density. Staining areas were quantified by image analysis and were expressed as the area of ZIKV staining divided by the area of DAPI staining for each organ/tissue. Data are presented for midguts, bodies (whole mosquitoes minus the area covered by midgut tissue), heads and salivary glands for mosquitoes that had infection in at least one tissue/organ. Post hoc comparisons of the main effect of days post infection on ZIKV staining density was carried out for each organ by Sidak’s method. Lines join comparisons where significant increases in ZIKV staining density had occurred for the main effect of days post infection. (C-E) High resolution images of ZIKV in infected mosquito midgut, head and salivary glands, respectively.

**Fig 5:**
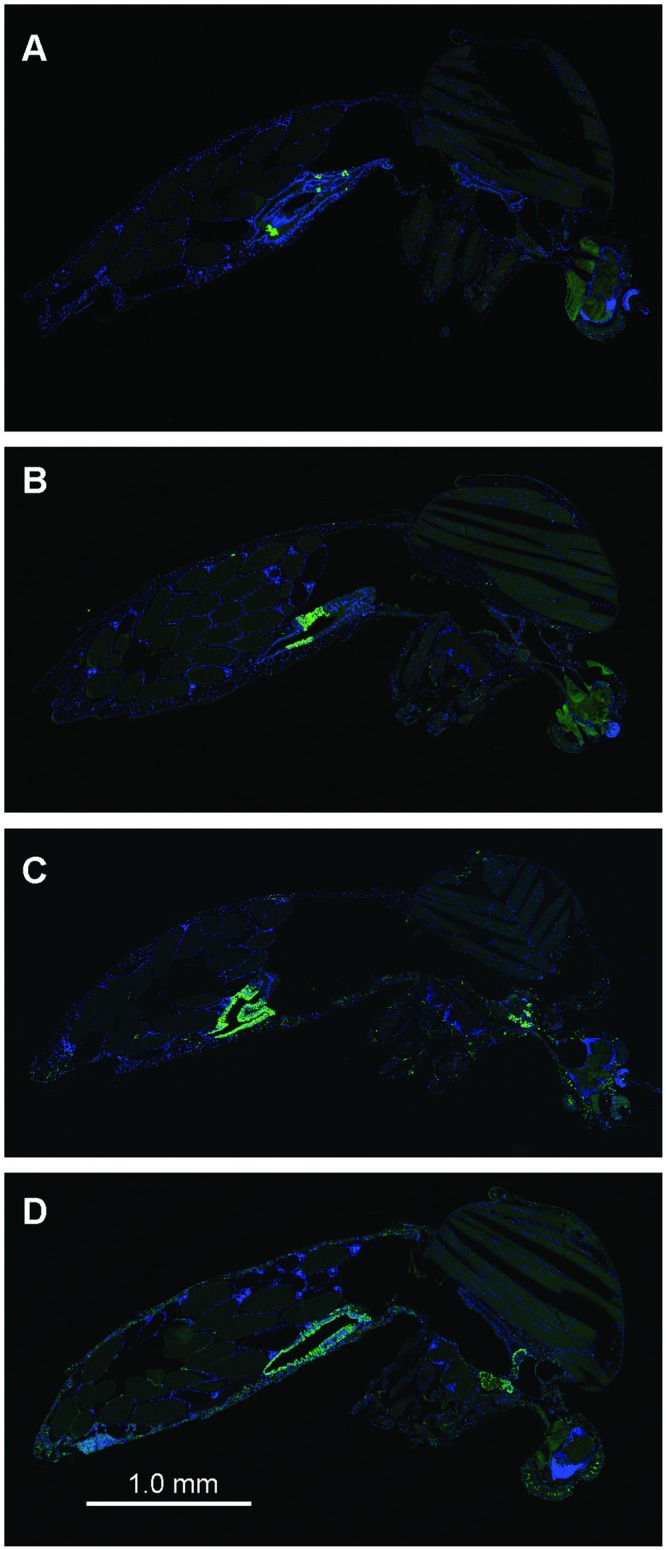
Immunofluorescence of whole *Ae*. *aegypti* mosquito sections. Whole *Ae*. *aegypti* mosquito sections showing infection and dissemination of ZIKV throughout mosquito tissues over a 14 day incubation period. Representative sections from different mosquitoes were selected at various time points. (A) 3 dpi (B) 7 dpi (C) 10 dpi and (D) 14 dpi. Immunofluorescence staining was performed as described for Fig 4. Green, ZIKV infection. Blue, DNA.

**Fig 6:**
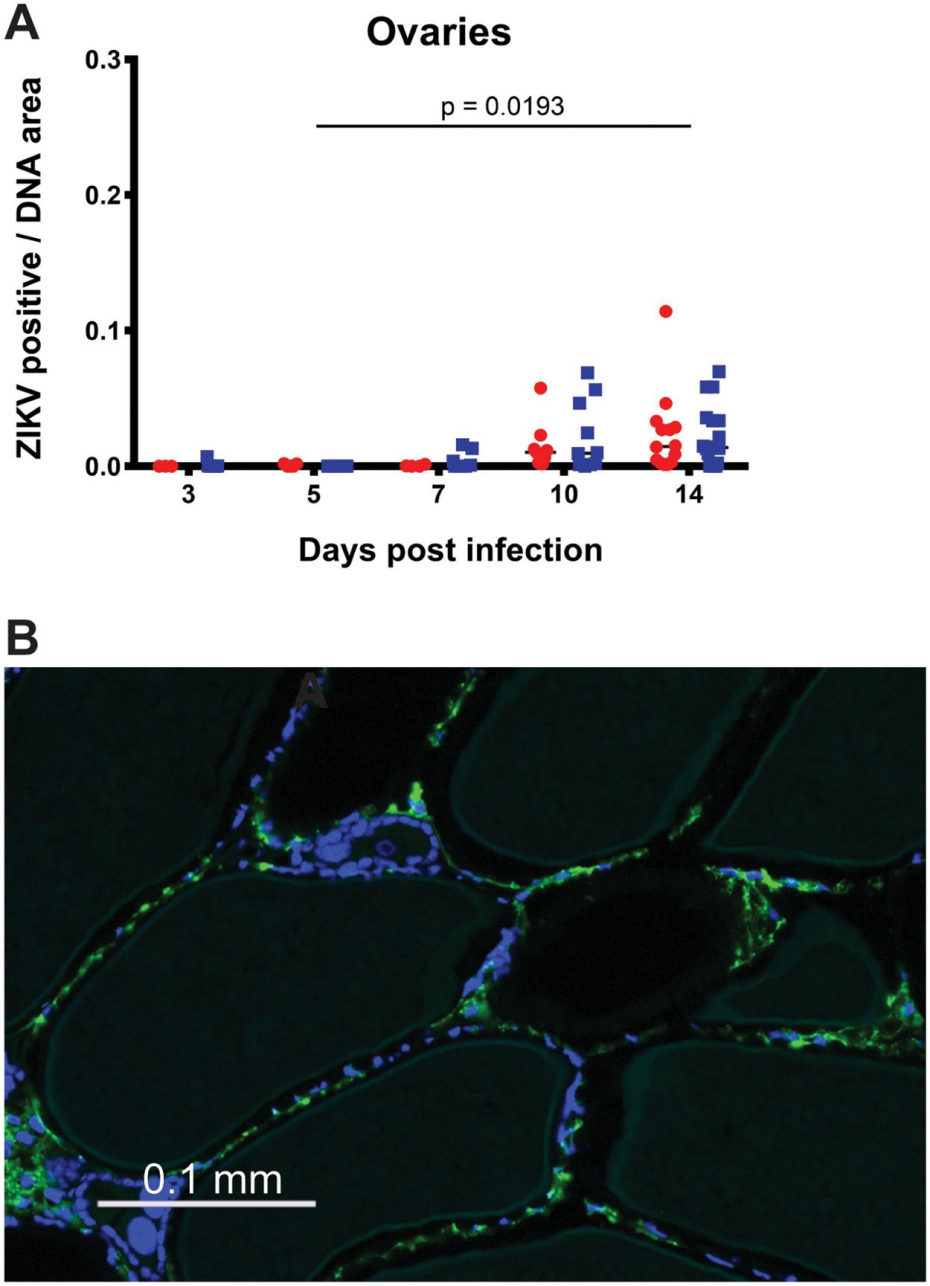
ZIKV infection in *Ae*. *aegypti* ovaries. (A) Quantification of ZIKV staining density relative to DNA over time, calculated as described for Fig 4. (B) High resolution image of ZIKV staining in the follicular epithelium of mature oocytes within *Ae*. *aegypti* ovaries. Green, ZIKV infection. Blue, DNA. Post hoc comparisons of the main effect of days post infection on ZIKV staining density was carried out for each organ by Sidak’s method. Lines join comparisons where significant increases in ZIKV staining density had occurred for the main effect of days post infection.

To visualize ZIKV distribution in *Ae*. *aegypti* tissues over time, we performed immunofluorescent antibody staining using a monoclonal antibody recognising *Flavivirus* NS1 proteins (Figs 4A and 5). We quantified ZIKV staining density (Fig 4B) through image analysis of the relative staining area of ZIKV to DNA for individual organs/tissues (Fig 4C–4E). The ZIKV staining density in mosquito midguts was visible from 3 dpi (Fig 4B). ZIKV staining was detectable in tissue/organs surrounding midguts (“body” samples) from 7 dpi. It was detected in the heads of a majority of mosquitoes from 10 dpi. Fewer salivary glands than other organs/tissues could be observed within the mid-sagittal mosquito sections, however, staining of the salivary glands that were observed indicated that a small proportion were infected by 7 dpi. By 10 dpi, all salivary glands had detectable ZIKV staining. An analysis of the staining density within the tissue/organs as a function of dpi and temperature regime found that, for all tissues, the effect of time post infection on ZIKV staining density was highly significant, whereas the effect of temperature regime was not significant (S3 Table). Interactions between time and temperature were non-significant in all cases. Post hoc comparisons revealed that significant increases in staining density occurred between 7 and 14 dpi for all tissues (Fig 4B). ZIKV was also detected and quantified within the ovaries of *Ae*. *aegypti* mosquitoes, which showed a significant increase in staining density between 5 and 14 d (Fig 6A and S3 Table). Staining was limited to the follicular epithelium surrounding oocytes (Fig 6B).

### Thresholds for transmission of ZIKV in mosquito saliva

We found that a disseminated titre of 7.50 log_10_ genome copies per mosquito wings/ legs (sensitivity of 0.943; 95% CI: 0.857–1.000) was required to predict successful infection of mosquito saliva in *Ae*. *aegypti*. Surprisingly, a lower threshold titre of 6.52 log_10_ (sensitivity of 0.922; 95% CI: 0.843–0.980) was necessary in *Ae*. *albopictus* to obtain ZIKV infection in saliva, in the proportions (2/20 each at 7 and 14 dpi in constant and 2/20 at 14 dpi in fluctuating temperature regimes) of mosquitoes that were able to transmit the virus.

## Discussion

Our study demonstrates that *Ae*. *aegypti* populations from north Queensland are susceptible to a Brazilian epidemic ZIKV strain from Asian lineage, and able to transmit ZIKV from 10 dpi. We also show that Torres Strait *Ae*. *albopictus* could be infected in high percentages, but only 10% could transmit virus by 14 days. Our results suggest that a high threshold titre of disseminated infection in *Ae*. *aegypti* was required to overcome the salivary barrier and allow transmission. A recent report suggested that a threshold viral load of at least 10^5.1^ TCID_50_ equivalents/mL in the legs and wings of Australian *Ae*. *aegypti* mosquitoes had to be reached for transmission of the prototype African strain of ZIKV to occur [58]. The infectious titre of disseminated virus could therefore be a significant predictor of virus detection in saliva. A similar correlation between disseminated virus titre and transmission rate has been reported for ZIKV [69] and DENV-1 [70], with high dissemination titres resulting in increased transmissibility by *Ae*. *aegypti*.

In Australia, variable vector competence of *Ae*. *aegypti* populations from north Queensland for Zika has been reported [48, 58, 59]. Those populations were shown to be competent vectors for the African lineage of ZIKV [58], but relatively inefficient vectors of a Western Pacific ZIKV strain belonging to the Asian ZIKV lineage [59]. Our data suggest that *Ae*. *aegypti* from northern Queensland in Australia may be less susceptible to Asian ZIKV strains than to the prototype African strain [58]. Our findings are supported by the results of oral challenges of Australian *Ae*. *aegypti* with a strain of ZIKV from the Western Pacific [59] in which infection and transmission rates were 40 and 37% respectively, using a similar virus titre to that employed here (8.5 and 8.8 log CCID_50_/ml, for the Western Pacific and Brazilian strains, respectively). It should be noted that the titres used in both studies are higher than those expected in typical human viremias [6, 36]. However, oral challenge of Australian *Ae*. *aegypti* with a lower titre of the Western Pacific ZIKV strain (5.6 log CCID_50_) resulted in only 3% of mosquitoes becoming infected [59]. Our study suggests that both high viremias and high disseminated threshold titres are required in order to obtain successful infection of *Ae*. *aegypti* and allow viral transmission to occur. Although *Ae*. *aegypti* could transmit ZIKV at moderate efficiency following challenge with a high titre, we have shown that under similar conditions, the transmission capability of Torres Strait *Ae*. *albopictus* was only 10%. *Ae*. *albopictus* is therefore less likely to participate in local transmission cycles than *Ae*. *Aegypti* in Australia.

A higher transmission rate (87%) of a Cambodian ZIKV strain (Asian lineage) has been reported for *Ae*. *aegypti* from Cairns in north Queensland [48]. Our data suggest the vector competence of Australian *Ae*. *aegypti* mosquitoes could depend on the geographical origin of populations and the virus strain/genotype, although differences between experiments will also contribute to the variation. The importance of investigating vector/virus strain interactions was recently demonstrated for a strain of *Ae*. *aegypti* from New Caledonia [69]. Infection, dissemination and transmission rates were significantly lower for recently isolated ZIKV strains from Africa and Asian lineages, compared with older African lineage isolates. In compatible combinations, ZIKV transmission occurred as early as 6 dpi [69]. Such genotype × genotype interactions have also been reported for DENV transmission [71]. Our study is in agreement with proportions of mosquitoes able to transmit ZIKV at 14 dpi reported for American *Ae*. *aegypti* challenged with Brazilian (75%), Puerto Rican (65%), and Malaysian (53%) ZIKV strains [72]. Similar to a study of French Polynesian *Ae*. *aegypti* [28], we found a significant increase in ZIKV transmission percentages from early time points (3 and 7 dpi) to 14 dpi. Similar transmission patterns have also been observed for other commonly investigated flaviviruses, i.e. dengue [73]. Our results from immunofluorescence analysis indicate that ZIKV transmission in *Ae*. *aegypti* potentially occurs from 10 dpi, similar to populations from the Island of Madeira in Portugal that were infectious at 9 days following an oral challenge with a New Caledonian ZIKV strain [49]. In contrast, *Ae*. *aegypti* mosquitoes from Singapore were able to transmit an Ugandan ZIKV strain as early as 5 dpi [74].

Compared to *Ae*. *aegypti*, *Ae*. *albopictus* mosquitoes were poor vectors for the Brazilian strain of ZIKV. The ZIKV transmission percentages observed in our study are similar to those reported for French and Italian *Ae*. *albopict*us mosquitoes challenged with ZIKV from the Asian genotype [49, 54]. However, the infection (10–18%) and disseminated infection (10–29%) rates reported in these studies were much lower than those observed in our study. Our results are strikingly different from a vector competence study in Singapore reporting that all *Ae*. *albopictus* mosquitoes challenged with an Ugandan ZIKV strain were infectious by 14 dpi [51]. *Ae*. *albopictus* populations from the Australian Torres Strait Islands have previously been shown to be highly susceptible to a Cambodian ZIKV strain, with a high prevalence (>75%) of virus in saliva at day 14 post-infection [48]. This suggests that the transmission of ZIKV in this population of *Ae*. *albopictus* is highly virus strain-dependent, as previously reported for American *Ae*. *albopictus* populations [57]. A specific vector/virus combination may therefore be more efficient at transmitting ZIKV than another.

The extrinsic incubation period, which is the time between oral infection and presence of virus in the saliva of vectors, is a major determinant of transmission efficiency [75]. We established the kinetics of ZIKV infection, dissemination and transmission in *Ae*. *aegypti* by measuring viral RNA in mosquito tissues and live virus in saliva and mosquito organs and tissues and measured viral RNA in *Ae*. *albopictus* tissues and live virus in saliva. Our findings support an extrinsic incubation period (EIP) of approximately 10 days in *Ae*. *aegypti* under the conditions tested. We found that there were dose-dependent thresholds for infection of salivary glands in both species. Surprisingly, despite the lower transmission percentages observed for *Ae*. *albopictus* compared to *Ae*. *aegypti*, the estimated threshold for transmission was also lower. The result suggests factors other than disseminated viral titre may be responsible for the transmission percentages observed in *Ae*. *albopictus*. Possible explanations for the lower ZIKV transmission percentages at 14 dpi for *Ae*. *albopictus*, compared to *Ae*. *aegypti*, is that EIP is longer in *Ae*. *albopictus*, and/or may be modulated significantly by temperature. This has important public health implications for preparedness, and efficient implementation of mosquito control efforts. A recent study reported that the administration of a second, non-infectious blood meal significantly shortened the EIP of ZIKV-infected *Ae*. *aegypti* and *Ae*. *albopictus* by enhancing virus escape from the mosquito midgut [76]. *Ae*. *albopictus* may therefore be more competent for ZIKV transmission under field conditions of frequent feeding, suggesting the risk of an outbreak mediated by this vector may be higher than is indicated by our data. Whether this holds true for Australian *Ae*. *aegypti* and *Ae*. *albopictus* remains to be determined. Last, we observed ZIKV staining in mosquito ovarian tissue, limited to the follicular epithelium surrounding developing eggs. This may indicate a potential route of infection leading to vertical transmission, which has been observed recently from field specimens collected as larvae [38].

Although most vector competence studies only take mean temperature values into account, recent evidence for DENV shows that diurnal temperature range (DTR) plays an important role in influencing the behaviour of *Ae*. *aegypti* [73, 77]. The DTR mimics more realistic field conditions, which could provide more accurate predictive disease outbreak models [73, 77, 78]. Taking into account the daily temperature fluctuation recorded during the summer months in Cairns Australia, we tested the effect of temperature fluctuations on *Ae*. *aegypti* and *Ae*. *albopictus* vector competence for ZIKV. Fluctuating temperature significantly affected viral dissemination to wings and legs rather than viral titre in bodies. Our findings suggest using a DTR that mimics field conditions is needed to better understand infection dynamics within mosquito hosts.

This study has demonstrated that north Queensland *Ae*. *aegypti* are more competent for a Brazilian strain of ZIKV than *Ae*. *albopictus*, confirming that *Ae*. *aegypti* is the primary vector of Asian lineage ZIKV. The risk of emergence of ZIKV in Australia is potentially high due to the presence of competent mosquito vectors, climatic conditions suitable for transmission, imported cases, and a large naïve population for ZIKV. However, our data were obtained under high-titre challenge conditions and should be viewed in the context of a recent study that shows low competence of north Queensland *Ae*. *aegypti* under more typical viremic titres [59]. We also need to add the caveat that our estimates of vector competence were derived from a single experimental replicate. Additional replicates may yield different estimates due to stochastic variance inherent in vector competence experiments.

In the absence of an effective vaccine and as ZIKV transmission is primarily vector-borne, mosquito control is likely to be the most effective preventative measure. In this regard, the use of the endosymbiotic bacterium *Wolbachia pipientis* has shown potential for the biocontrol of ZIKV [79] and other human pathogenic flaviviruses and alphaviruses [80, 81]. Large field releases in north Queensland of novel *Wolbachia*-transinfected *Ae*. *aegypti* mosquitoes, refractory to infection by a range of arboviruses [79–81], have shown the ability to drive *Wolbachia* into wild populations [82]. Our data could be beneficial for modelling likely ZIKV transmission dynamics in north Queensland and addressing emerging ZIKV threats to Australia.

## Supporting information

S1 Table ZIKV titres in *Ae*. *aegypti* and *Ae*. *albopictus* bodies. Number of RNA viral copies detected in the bodies of *Ae*. *aegypti* and *Ae*. *albopictus* maintained at 28°C constant or fluctuating temperature conditions.
(DOCX)

S2 Table ZIKV titres in *Ae*. *aegypti* and *Ae*. *albopictus* wings and legs. Number of RNA viral copies detected in the wings and legs of *Ae*. *aegypti* and *Ae*. *albopictus* maintained at 28°C constant or fluctuating temperature conditions.
(DOCX)

S3 Table Summary of the statistical significance of factors affecting ZIKV staining density in different mosquito tissues. The effects of days post infection, temperature regime and their interaction on ZIKV staining density were examined for different mosquito tissue by two-way ANOVA.
(DOCX)
