# Supplementary material for: Vector competence of Australian Aedes aegypti and Aedes albopictus for an epidemic strain of Zika virus
Source: PLoS Negl Trop Dis. 2019 Apr 4;13(4):e0007281. doi: 10.1371/journal.pntd.0007281 (PMC6467424; doi:10.1371/journal.pntd.0007281)
Supplement: S1 Table — Number of RNA viral copies detected in the bodies of Ae. aegypti and Ae. albopictus maintained at 28°C constant or fluctuating temperature conditions. (DOCX) [file pntd.0007281.s001.docx]

|  | | Constant Temperature | | Fluctuating Temperature | |
| --- | --- | --- | --- | --- | --- |
| Species | dpi | Median | Interquartile range | Median | Interquartile range |
|  |  |  |  |  |  |
| *Ae. aegypti* | 3 | 1.1×10^6^ | 3.3×10^5^-1.9×10^6^ | 2.5×10^6^ | 6.6×10^5^-4.9×10^6^ |
|  | 7 | 3.8×10^7^ | 1.2×10^5^-6.7×10^7^ | 4.9×10^7^ | 1.8×10^7^-8.8×10^7^ |
|  | 14 | 3.2×10^8^ | 2.5×10^8^-4.7×10^8^ | 1.0×10^8^ | 6.3×10^7^-2.6×10^8^ |
| *Ae. albopictus* | 3 | 3.3×10^5^ | 1.8×10^5^-4.8×10^5^ | 4.0×10^5^ | 2.1×10^5^-7.1×10^5^ |
|  | 7 | 2.1×10^7^ | 1.0×10^7^-5.9×10^7^ | 1.2×10^7^ | 5.4×10^6^-2.0×10^7^ |
|  | 14 | 6.1×10^7^ | 3.7×10^7^-1.4×10^8^ | 8.5×10^7^ | 3.7×10^7^-2.1×10^8^ |
