# Supplementary material for: Vector competence of Australian Aedes aegypti and Aedes albopictus for an epidemic strain of Zika virus
Source: PLoS Negl Trop Dis. 2019 Apr 4;13(4):e0007281. doi: 10.1371/journal.pntd.0007281 (PMC6467424; doi:10.1371/journal.pntd.0007281)
Supplement: S2 Table — Number of RNA viral copies detected in the wings and legs of Ae. aegypti and Ae. albopictus maintained at 28°C constant or fluctuating temperature conditions. (DOCX) [file pntd.0007281.s002.docx]

|  | | 28°C Constant Temperature | | 28°C Fluctuating Temperature | |
| --- | --- | --- | --- | --- | --- |
| Species | dpi | Median | Interquartile range | Median | Interquartile range |
|  |  |  |  |  |  |
| *Ae. aegypti* | 3 | 1.5×10^4^ | 1.3×10^4^-1.7×10^4^ | 9.1×10^4^ | 8.0×10^4^-1.0×10^5^ |
|  | 7 | 1.2×10^5^ | 2.9×10^4^-2.0×10^5^ | 1.6×10^4^ | 5.4×10^3^-1.0×10^6^ |
|  | 14 | 2.5×10^8^ | 1.2×10^8^-4.6×10^8^ | 5.4×10^7^ | 3.6×10^7^-7.0×10^7^ |
| *Ae. albopictus* | 3 | 1.2×10^4^ | 7.5×10^3^-1.3×10^4^ | ND | ND |
|  | 7 | 4.3×10^4^ | 1.9×10^4^-2.0×10^5^ | 1.4×10^4^ | 1.2×10^4^-1.6×10^5^ |
|  | 14 | 1.6×10^6^ | 3.2×10^4^-6.8×10^6^ | 9.9×10^4^ | 6.3×10^4^-9.0×10^6^ |

Abbreviations: ND, not detected
