# Supplementary material for: Vector competence of Australian Aedes aegypti and Aedes albopictus for an epidemic strain of Zika virus
Source: PLoS Negl Trop Dis. 2019 Apr 4;13(4):e0007281. doi: 10.1371/journal.pntd.0007281 (PMC6467424; doi:10.1371/journal.pntd.0007281)
Supplement: S3 Table — The effects of days post infection, temperature regime and their interaction on ZIKV staining density were examined for different mosquito tissue by two-way ANOVA. (DOCX) [file pntd.0007281.s003.docx]

|  | Source of variation | | |
| --- | --- | --- | --- |
| Tissue | Days post infection | Temperature | Interaction |
|  |  |  |  |
| Midguts | *p*<0.0001 | *p*=0.2680 | *p*=0.5002 |
| Bodies | *p*<0.0001 | *p*=0.5382 | *p*=0.9779 |
| Heads | *p*<0.0001 | *p*=0.5949 | *p*=0.6508 |
| Salivary glands | *p*<0.0001 | *p*=0.7082 | *p*=0.9621 |
| Ovaries | *p*=0.0013 | *p*=0.6838 | *p*=0.9229 |
